# Putative Periodontal Pathogens, *Filifactor alocis* and *Peptoanaerobacter stomatis,* Induce Differential Cytokine and Chemokine Production by Human Neutrophils

**DOI:** 10.3390/pathogens8020059

**Published:** 2019-05-01

**Authors:** Aruna Vashishta, Emeri Jimenez-Flores, Christopher K. Klaes, Shifu Tian, Irina Miralda, Richard J. Lamont, Silvia M. Uriarte

**Affiliations:** 1 Department of Medicine, School of Medicine, University of Louisville, 570 S. Preston St., Louisville, KY 40202, USA; aruna.vashishta@louisville.edu (A.V.); emeri.jimenez@upr.edu (E.J.-F.); keith.klaes@louisville.edu (C.K.K.); shifu.tian@pennmedicine.upenn.edu (S.T.); 2 Department of Oral Immunology and Infectious Diseases, School of Dentistry, University of Louisville, 570 S. Preston St., Louisville, KY 40202, USA; rich.lamont@louisville.edu; 3 Department of Microbiology & Immunology, School of Medicine, University of Louisville, 505 S. Hancock St., Louisville, KY 40202, USA; irina.miralda@louisville.edu

**Keywords:** Emerging oral pathogens, human neutrophils, cytokines and chemokines, chemotaxis

## Abstract

Periodontitis is a highly prevalent infectious disease that affects ~ 50% of the adults in the USA alone. Two Gram-positive anaerobic oral bacteria, *Filifactor alocis* and *Peptoanaerobacter* *stomatis*, have emerged as important periodontal pathogens. Neutrophils are a major component of the innate host response in the gingival tissue, and the contribution of neutrophil-derived cytokines and chemokines plays a central role in disease progression. The pattern of cytokines and chemokines released by human neutrophils upon stimulation with newly appreciated periodontal bacteria compared to the keystone oral pathogen *Porphyromonas gingivalis* was investigated. Our results showed that both *F. alocis* and *P. stomatis* triggered TLR2/6 activation. *F. alocis* induced significant changes in gene expression of cytokines and chemokines in human neutrophils compared to unstimulated cells. However, except for IL-1ra, neutrophils released lower levels of cytokines and chemokines in response to *F. alocis* compared to *P. stomatis.* Furthermore, bacteria-free conditioned supernatant collected from neutrophils challenged with *P. stomatis*, but not from *P. gingivalis* or *F. alocis,* was chemotactic towards both neutrophils and monocytes. Elucidating stimuli-specific modulation of human neutrophil effector functions in the context of dysbiotic microbial community constituents provides valuable information for understanding the pathogenesis of periodontal diseases.

## 1. Introduction

Periodontitis is a complex polymicrobial chronic inflammatory infectious disease that affects approximately 50% of the adults- older than 30 years of age- in the USA [1]. The periodontium, the tissue that surrounds and supports the tooth, is severely damaged as a consequence of the chronic unresolved inflammation promoting an increase rate of bone resorption and loss of the tooth [2]. The etiology of periodontitis involves an imbalance between the indigenous microbial community and the host which promotes a destructive cyclic inflammation which facilitates the growth of pathogenic organisms [3,4]. Analysis of dental plaque composition through advanced high-throughput methodologies has revealed the presence of a large number of emerging oral pathogens in disease sites as compared to healthy sites [5,6,7]. *Filifactor alocis* and *Peptoanaerobacter stomatis*, are two Gram-positive examples of putative oral pathogens associated with periodontitis, dentoalveolar abscesses and endodontic infections [8,9,10,11]. Furthermore, inclusion of *F. alocis* as a diagnostic indicator of disease has been proposed [12]. 

Regarding the pathogenic potential of *F. alocis,* it has been established that the organism can invade gingival epithelial cells, produces trypsin-like proteases, and resists oxidative stress [13,14,15]. In addition, *F. alocis* can form biofilms in vivo, preferentially colonizing the apical parts of the gingival pocket in close proximity to the soft tissues [16]. Moreover, in vivo studies using the mouse subcutaneous chamber model show that *F. alocis* is able to establish a local infection, which is resolved by 72 h, but also has the ability to spread to remote tissues such as spleen, lung and kidney causing acute kidney injury [17].

Neutrophils are the first innate immune cell to respond and be recruited in vast numbers to the site of infection [18,19]. They enter the periodontal pocket as part of the host response to combat the microbial challenge and to maintain homeostasis in the oral cavity [20]. However, in periodontitis, the dysbiotic microbial pathogens are able to withstand neutrophil potent antimicrobial mechanisms perpetrating a chronic inflammatory environment which benefits the oral pathogenic community [3,19]. 

Non-opsonized and serum opsonized *F. alocis* is effectively internalized by human neutrophils. However, *F. alocis* remains viable 6 hours post challenge by inducing minimal respiratory burst response and preventing phagosome maturation in human neutrophils [21]. In contrast, *P. stomatis* is poorly internalized by human neutrophils but effectively killed once inside a phagosome. However, 80% of *P. stomatis* initial inoculum, which is not phagocytized, remains viable up to 2 hours post challenge and killed primarily by oxygen independent mechanisms [22]. In addition to playing a relevant role in microbial killing, neutrophils also orchestrate the immune response by contributing to the cytokine and chemokine pool during inflammation. It is well established that neutrophils have the capacity to transcribe and synthesize de novo cytokines and chemokines [23]. Since they are the first cells recruited to an inflammatory site in high number, neutrophils contribution to the cytokine and chemokine pool becomes very relevant in the modulation of the immune response [24,25]. Neutrophils ability to store cytokines and chemokines in their granules, is advantageous compared to other leukocytes since it ensures quick release of inflammatory mediators at the site of inflammation [25]. Both *F. alocis* and *P. stomatis* induce secretory vesicle, gelatinase granule and specific granule exocytosis but only *P. stomatis* is able to mobilize azurophilic granule exocytosis [21,22,26]. 

Stimulation of human neutrophils with different bacterial components or with whole organisms will result in release of different types of inflammatory mediators [27,28]. The main goal of this study was to determine the expression and release of human neutrophil-derived cytokines and chemokines induced by *F. alocis* stimulation. Furthermore, the release of different cytokines and chemokines after stimulation with *F. alocis*, *P. stomatis*, and the keystone oral pathogen—*Porphyromonas gingivalis*—was determined. Our results show that although *F. alocis* stimulation induces both the expression and release of several neutrophil-derived cytokines and chemokines; *P. stomatis* stimulation triggers higher release of these inflammatory mediators sufficient to induce chemotaxis of both neutrophils and monocytes. 

## 2. Results

### 2.1. *F. alocis* Challenge Induced Both Expression and Release of Neutrophil-Derived Cytokines and Chemokines

**Figure 1:**
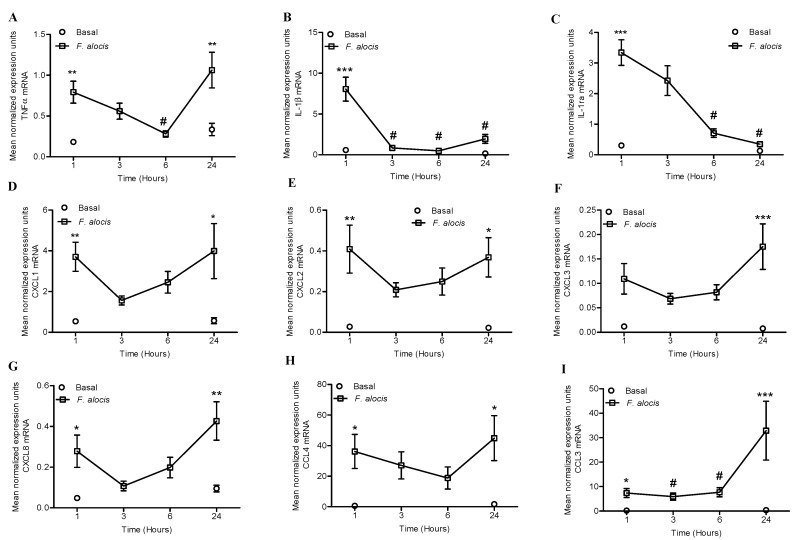
*F. alocis* challenge induced the mRNA expression of cytokines and chemokines in human neutrophils. Neutrophils were unstimulated (Basal), or challenged with *F. alocis* (multiplicity of infection (MOI) 10:1) for 1-3-6-24h and mRNA expression determined by RT-qPCR. (**A**) TNFα mRNA expression, (**B**) IL-1β mRNA expression, (**C**) IL-1ra mRNA expression, (**D**) CXCL1 mRNA expression, (**E**) CXCL2 mRNA expression, (**F**) CXCL3 mRNA expression, (**G**) CXCL8 mRNA expression, (**H**) CCL4 mRNA expression, (**I**) CCL3 mRNA expression. The data are shown as mean normalized expression units after GAPDH mRNA normalization ± SEM of n = 5–7 separate experiments. * *p* < 0.05, ** *p* < 0.01, *** *p* < 0.001 compared to the corresponding basal time point at 1 h or 24 h. (**A**, **B, C**) # *p* < 0.05 compared to 1 h. (**I**) # *p* < 0.05 compared to 24 h.

We wanted to determine if challenge of human neutrophils with *F. alocis* would induce changes in the gene expression of neutrophil-derived cytokines and chemokines. The kinetics of TNFα, IL-1β, IL-1receptor antagonist (IL-1ra), CXCL1, CXCL2, CXCL3, CXCL8, CCL3, and CCL4 mRNA expression were determined by RT-qPCR. As shown in Figure 1, *F. alocis* induced a significant increase in the mRNA expression of all the transcripts, except for CXCL3, by 1 h post challenge compared to unstimulated cells. The mRNA expression of TNFα peaked at 1 h post *F. alocis* challenge, followed by a significant decrease by 6 h; a trend that was reversed by 24 h showing a significant increase compared to unstimulated cells (Figure 1A). In contrast, the mRNA expression of both IL-1β and its inhibitor IL-1ra, peaked by 1 h post *F. alocis* challenge and showed a time dependent decrease to baseline levels by 24 h (Figure 1B,C). The four CXCL chemokines, CXCL1-CXCL2-CXCL3-CXCL8, and the CCL chemokine -CCL4- showed a similar mRNA time-course pattern, albeit with different expression levels, showing a peak by 1 h post *F. alocis* challenge which decreased by 3 and 6 h but showed a significant increase with maximum expression for CXCL3 and CXCL8 by 24 h compared to unstimulated cells (Figure 1D–H). In contrast to all the other cytokines and chemokines, CCL3 was the only chemokine that showed a time dependent increase in its mRNA expression, reaching the maximum expression by 24 h post *F. alocis* challenge (Figure 1I).

Next, we determined whether challenge of human neutrophils with *F. alocis* would induce the release of cytokines, CXCL, and CCL chemokines. This was measured in neutrophil-derived supernatants collected from unstimulated cells or after 24 h of challenge with either non-opsonized or serum opsonized *F. alocis* at a multiplicity of infection (MOI) of 10:1. Table 1 shows that both non-opsonized or serum opsonized *F. alocis* induced significant release of pro-inflammatory cytokines TNFα, IL-1β as well as the anti-inflammatory cytokine IL-1ra when compared to unstimulated neutrophils. Similarly, significant levels of CXCL1 and CXCL8 –both potent neutrophil chemokines- were released by *F. alocis* independent of opsonization. Release of CCL3 and CCL4 -both potent monocyte chemokines- was increased by *F. alocis* challenge; however, only the levels induced by the opsonized bacteria reached statistical significance compared to unstimulated cells. Overall, challenge with either non-opsonized or serum opsonized *F. alocis* showed a similar profile of cytokine and chemokine release. For all further experiments, the non-opsonized condition was used. 

### 2.2. Distinct Pro-Inflammatory and Anti-Inflammatory Cytokine Release by *F. alocis* Compared to *P. gingivalis* and *P. stomatis*

**Figure 2:**
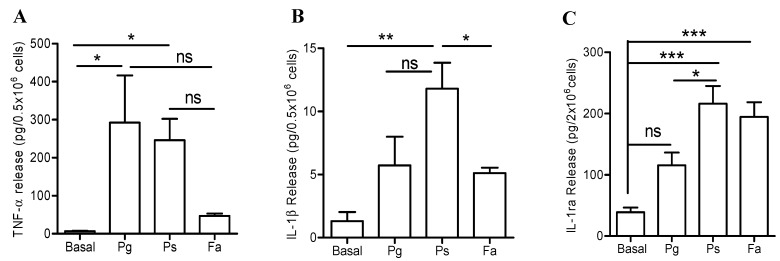
Differential release of pro and anti-inflammatory cytokines by neutrophils when challenge with three oral bacteria. Neutrophils were unstimulated (basal), challenged with *P. gingivalis* (Pg), *P. stomatis* (Ps), or *F. alocis* (Fa) at MOI 10. Bacteria- and cell- free supernatants were collected after 24 h and cytokine levels measured by ELISA or Milliplex Luminex. (**A**) Data are expressed as mean ± SEM of TNFα release from 5 independent experiments. (**B**) Data are expressed as mean ± SEM of IL-1β release from 5 independent experiments. (**C**) Data are expressed as mean ± SEM of IL-1ra release from 6 independent experiments.* *p* < 0.05, ** *p* < 0.01, *** *p* < 0.001, ns = non-significant.

Our data thus far showed that *F. alocis* challenge of human neutrophils induced changes in gene expression and triggered release of both pro-inflammatory cytokines, TNFα and IL-1β, as well the anti-inflammatory cytokine, IL-1ra. However, the magnitude and release of cytokines and chemokines by neutrophils is tailored to the stimulation they encounter [27]. Next we sought to compare the pro and anti-inflammatory cytokine release profile induced by *F. alocis* to the response elicited by the consensus keystone periodontal pathogen, *P. gingivalis*, and another emerging oral pathogen *P. stomatis*. Released levels of neutrophil-derived pro and anti-inflammatory cytokines were measured in the supernatant collected after 24 h of bacterial challenge. Higher levels of TNFα were detected 24 h after *P. gingivalis* and *P. stomatis* challenge compared to *F. alocis* (Figure 2A). Furthermore, *P. stomatis* challenge induced a significantly higher release of IL-1β compared *F. alocis* stimulation (Figure 2B). Unlike *P. gingivalis*, stimulation with either *F. alocis* or *P. stomatis* resulted in significant release of the anti-inflammatory cytokine, IL-1ra by human neutrophils compared to unstimulated cells (Figure 2C). These data indicates a differential modulation of neutrophil-derived cytokines by the three oral bacteria, with only *P. stomatis* inducing significant release of TNFα, IL-1β and IL-1ra. 

### 2.3. Both *F. alocis* and *P. stomatis* Activated TLR2/6 to a Greater Extent than TLR2/1

**Figure 3:**
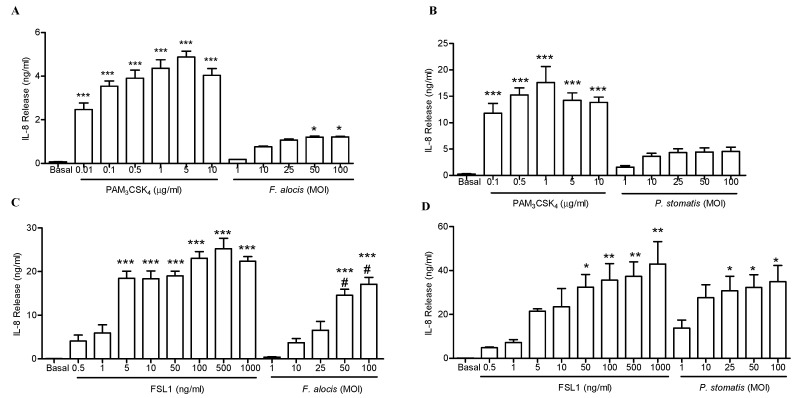
*F. alocis* and *P. stomatis* challenge induced enhanced Toll-like receptor (TLR)2/6 activation compared to TLR2/1. (**A**,**B**) HEK293-TLR2/1 cells were unstimulated (Basal), stimulated with increasing concentrations of TLR2/1 ligand PAM_3_CSK_4_, increasing multiplicity of infection (MOI) of *F. alocis*, increasing MOI of *P. stomatis* for 24 h. (**C**,**D**) HEK293-TLR2/6 cells were unstimulated (Basal), stimulated with increasing concentrations of TLR2/6 ligand FSL1, increasing MOI of *F. alocis*, increasing MOI of *P. stomatis* for 24 h. Bacteria- and cell-free supernatants were collected after 24 h stimulation, and IL-8 levels measured by ELISA. Data are expressed as mean ± SEM of ng/mL of IL-8 release from 3 separate experiments run in duplicate. * *p* < 0.05, ** *p* < 0.01, *** *p* < 0.001 compared to basal. # *p* < 0.001 compared to *F. alocis* MOI 1-10-25.

Both immune and non-immune cells possess a wide repertoire of microbe recognition receptors such as the Toll-like receptor (TLR) family. With the exception of TLR3 and TLR7, the rest of the TLR family members are expressed in human neutrophils [29]. Activation of TLRs leads to activation of transcription factors such as NF-kB and transcription of cytokines and chemokines [23]. *F. alocis* interaction with human neutrophils induces MAPK activation and neutrophil granule exocytosis in a TLR2-dependent manner [26]. However, upon activation TLR2 can form heterodimers with TLR1 or TLR6. *P. gingivalis* activates TLR2/1 signaling and promotes the crosstalk with other receptors such as complement receptor C5a and CR3 to evade killing and sustain inflammation [30,31]. Which type of TLR2 heterodimer combination can be activated by *F. alocis* and *P. stomatis* is not known. Our data shows a differential release of cytokines triggered by *F. alocis* and *P. stomatis* challenge of human neutrophils, so we next sought to determine which TLR2 heterodimer activation, either TLR2/1 or TLR2/6, will be induced by the putative oral pathogens. Human embryonic kidney cell lines (HEK293) that are stably transfected to express TLR2/1 or TLR2/6 heterodimers were challenged with increasing concentrations of *F. alocis* or *P. stomatis*. Activation of specific TLR was determined by measuring IL-8 production. Stimulation of HEK293-TLR2/1 cells with increasing concentrations of *F. alocis* or *P. stomatis,* from 1 to 100, resulted in minimal TLR2/1 stimulatory activity (Figure 3A,B). Contrastingly, stimulation of HEK293-TLR2/1 cells with the lowest concentration of its ligand, PAM_3_CSK_4_, resulted in significantly higher stimulatory activity compared to any of the doses tested with either *F. alocis* or *P. stomatis*. In contrast, Figure 3C shows a dose dependent increase in TLR2/6 activation by *F. alocis*. Similarly, *P. stomatis* induced significant TLR2/6 activation, but reaching a plateau in its response by MOI 10 (Figure 3D). These results suggest that both *F. alocis* and *P. stomatis* induced a stronger TLR2/6 activation compared to TLR2/1. 

### 2.4. Higher Release of CXCL1 and CCL Chemokines by Neutrophils when Challenged with *P. stomatis* Compared to *F. alocis* or *P. gingivalis*

**Figure 4:**
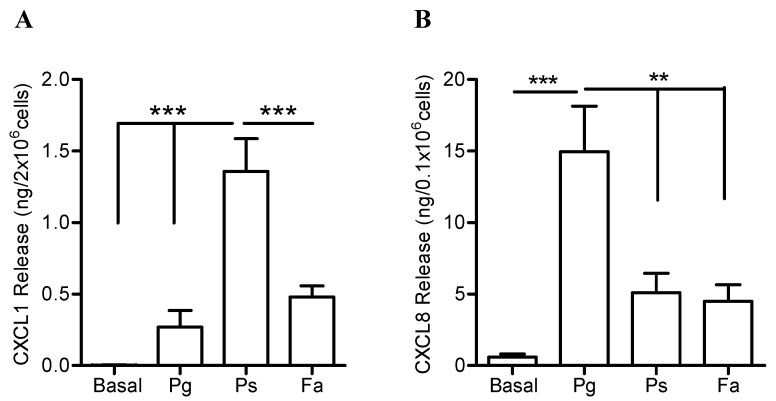
Differential release of CXCL chemokines by neutrophils when challenged with three oral bacteria. Neutrophils were unstimulated (basal), challenged with *P. gingivalis* (Pg), *P. stomatis* (Ps), *F. alocis* (Fa). MOI of 10 bacteria per neutrophil was used. Bacteria and cell free supernatants were collected after 24 h and cytokine levels measured by ELISA (CXCL8) or Milliplex Luminex (CXCL1). (**A**) Data are expressed as mean ± SEM of CXCL1 release from 7 independent experiments. (**B**) Data are expressed as mean ± SEM of CXCL8 release from 5 independent experiments. ** *p* < 0.01, *** *p* < 0.001.

The differential release of neutrophil cytokines triggered by the challenge with the three oral bacteria prompt us to investigate if similar differences would be observed in the release of the CXCL and CCL chemokines. First, levels of CXCL1 and CXCL8—two potent neutrophil chemokines—were determined 24 h post bacterial challenge. Human neutrophils exposed to *P. stomatis* released significantly higher levels of CXCL1 compared to the response elicited by *P. gingivalis* or *F. alocis* (Figure 4A). In contrast, *P. gingivalis* challenge induced significantly higher release of CXCL8 compared to *P. stomatis* and *F. alocis* (Figure 4B). Even though *F. alocis* challenge triggered the release of significantly higher levels of both CXCL1 and CXCL8 compared to basal conditions (Table 1); its secretion was significantly lower compared to the responses triggered by *P. stomatis* or *P. gingivalis*, respectively (Figure 4A,B).

**Figure 5:**
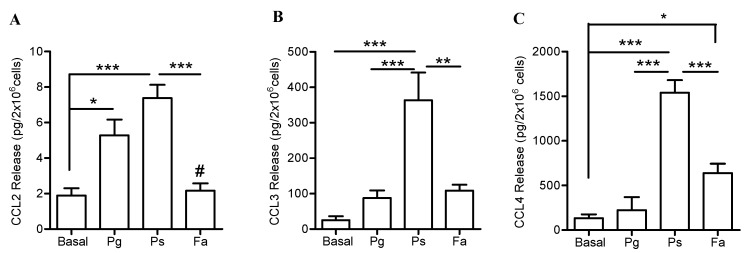
Differential release of CCL chemokines by neutrophils when challenged with three oral bacteria. Neutrophils were unstimulated (basal), challenged with *P. gingivalis* (Pg), *P. stomatis* (Ps), *F. alocis* (Fa). MOI of 10 bacteria per neutrophil was used. Bacteria- and cell- free supernatants were collected after 24 h and cytokine levels measured by Milliplex Luminex. (**A**) Data are expressed as mean ± SEM of CCL2 release from 5 independent experiments. (**B**) Data are expressed as mean ± SEM of CCL3 release from 4 independent experiments. (**C**) Data are expressed as mean ± SEM of CCL4 release from 5 independent experiments. * *p* < 0.05, ** *p* < 0.01, *** *p* < 0.001. In (**A**) # *p* < 0.05 compared to Pg.

Next, levels of CCL family of chemokines -CCL2-CCL3-CCL4- all of which induce monocyte recruitment, were determined 24 h post challenge by the three oral bacteria. Among these bacterial stimuli, only *P. stomatis* induced the release of significantly higher levels of all three CCL chemokines by neutrophils (Figure 5). *P. gingivalis-*stimulated neutrophils released higher levels of CCL2 compared to *F. alocis* or unstimulated cells (Figure 5A). However, *P. gingivalis* challenge induced minimal release of CCL3 (Figure 5B) and CCL4 (Figure 5C) by human neutrophils. *F. alocis* induced minimal release of CCL2 and CCL3 but stimulated significantly higher release of CCL4 compared to unstimulated cells (Figure 5C). Overall, these data show that only *P. stomatis* challenge strongly promotes the release of CXCL1, CCL2, CCL3, and CCL4 chemokines by human neutrophils. 

### 2.5. Release of Neutrophil-Derived Chemokines by *P. stomatis* Challenge Promotes Leukocyte Migration

**Figure 6:**
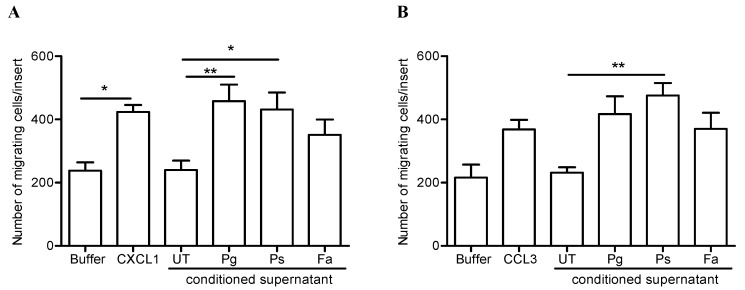
Supernatants collected from *P. stomatis* challenge have chemotactic activity for both neutrophils and monocytes. (**A**) Human neutrophils and in (**B**) monocytes were placed in the upper chamber of the transwell insert and 600 µL of buffer, CXCL1 (10 nM, a neutrophil chemoattractant), CCL3 (100 ng/mL, a monocyte chemoattractant), neutrophil-derived cell/bacteria-free conditioned supernatant collected after 24 h from unstimulated (UT), stimulated with *P. gingivalis* (Pg), *P. stomatis* (Ps), or *F. alocis* (Fa); were placed in the lower chamber. For neutrophil (**A**) or monocytes (**B**) chemotaxis the corresponding inserts were incubated at 37 °C, 5% CO_2_ incubator for 30 min or 120 min, respectively. Chemotaxis was assessed by light microscopic examination (magnification x100) of the underside of the membrane. The average number of cells from a total of 10 high-power fields was determined and data expressed as mean ± SEM of number of migrating cells/insert. (**A**), n = 7 independent experiments; (**B**), n = 4 independent experiments. * *p* < 0.05, ** *p* < 0.01.

To determine the biological activities of the neutrophil-derived chemokines induced by the different oral pathogens, chemotaxis assays were performed. First we sought to measure the chemotaxis of naïve neutrophils towards the cell-free supernatants collected after 24 h from unstimulated or from neutrophils challenged with *P. gingivalis*, *P. stomatis*, or *F. alocis*. The conditioned supernatants collected from both *P. gingivalis* and *P. stomatis* challenge showed significant chemotactic activity for human neutrophils compared to the response elicited by the supernatants collected from unstimulated cells (Figure 6A). The chemotactic response elicited by the supernatants collected from *P. gingivalis* and *P. stomatis* displayed a similar response as the potent neutrophil chemoattractant, CXCL1 (Figure 6A). In contrast, the conditioned supernatant collected from *F. alocis* challenge had no significant chemotactic activity for naïve neutrophils. 

Next, the chemotactic activity of human monocytes towards the conditioned supernatants from human neutrophils was tested. Among the three bacterial stimuli tested, only the conditioned supernatant collected from *P. stomatis* challenge exerted a significant chemotactic activity for human monocytes (Figure 6B). A chemotactic response that showed a higher trend compared to the response elicited by the recombinant CCL3 chemokine. In contrast, neither the conditioned supernatant collected from *P. gingivalis* nor *F. alocis* challenge induced significant chemotaxis of monocytes (Figure 6B). In summary, these results show that *P. stomatis* interaction with neutrophils results in the release of biologically active neutrophil-derived chemokines which induce chemotaxis of both neutrophils and monocytes. 

## 3. Discussion

Neutrophils are present in periodontal tissues both in health and in disease conditions [32]. Periodontitis is characterized by uncontrolled inflammation with dysregulated recruitment of neutrophils that fail to control the dysbiotic microbial community. Hence, it is of relevance to characterize how periodontal bacteria manipulate neutrophil effector functions. The production and release of cytokines and chemokines by neutrophils is stimuli dependent and finely tuned [33]. In the present study we report for the first time, to our knowledge, the production and release of human neutrophil-derived cytokines and chemokines in response to *F. alocis*. Furthermore, we compared the release of neutrophil-derived cytokines and chemokines induced by the two putative oral pathogens, *F. alocis* and *P. stomatis,* and by *P. gingivalis*. Our results, showed that among the three oral bacteria tested, *P. stomatis* induced greater release of biologically active neutrophil-derived chemokines to promote neutrophil and monocyte chemotaxis. 

A few studies have addressed neutrophil production and release of cytokines and chemokines to oral bacteria-derived agonist. Stimulation of human neutrophils with LPS from the oral pathogens, *Fusobacterium nucleatum* and *Aggregatibacter actinomycetemcomitans,* as well as from *E. coli* resulted in significantly higher levels of IL-1β, TNFα and IL-8 compared to unstimulated cells or the response elicited by LPS stimulation from *P. gingivalis* [34]. In addition, stimulation of neutrophils with *A. actinomycetemcomitans*-LPS and *E. coli*–LPS resulted in higher levels of the anti-inflammatory cytokine, IL-1ra, compared to stimulation with *P. gingivalis*-LPS in supernatants collected after 18 hours [34]. Another study showed that when peripheral blood neutrophils isolated from patients with active periodontitis are exposed to heat-killed *P. gingivalis* or *F. nucleatum,* they release significantly higher levels of pro-inflammatory cytokines compared to neutrophils from healthy donors [35]. Moreover, *in vitro* challenge of human neutrophils with heat-killed *F. nucleatum* induced significant upregulation of CXCL1, CXCL2, and CXCL3 mRNA [36]. Our findings confirm some of the earlier reports with LPS and heat-killed bacteria, and expand our knowledge of oral pathogens responses by showing that live *P. gingivalis* induced higher release of IL-8 from neutrophils compared to *F. alocis* and *P. stomatis*. Several different classes of stimuli, including LPS from oral bacteria, are known to induce the release of IL-1β; and can also modulate the production and release of IL-1ra [33,34,37]. Similar to the reports with *P. gingivalis*-LPS, the live organism did not induce significant release of IL-1β by human neutrophils. Additionally, *P. gingivalis* was a poor inducer of IL-1ra release compared to both *F. alocis* and *P. stomatis*. These results support the established role described for *P. gingivalis* as a manipulator of immune cells that can evade killing without jeopardizing the production of pro-inflammatory mediators [31,38]. 

In the present study, the neutrophil-derived supernatants collected after 24 h of *F. alocis* stimulation did not induce chemotaxis of naïve neutrophils or monocytes. However, we previously reported that human neutrophils infected with live or heat-killed *F. alocis*, display enhanced chemotaxis towards IL-8 compared to uninfected cells [26]. Combining the results from our previous and current study, we can propose that *F. alocis* manipulates neutrophils by enhancing their chemotactic capacity but does not induce a robust release of neutrophil-derived pro-inflammatory cytokines and chemokines. In contrast, the experiments performed with *P. stomatis* revealed that this oral pathogen induces robust release of neutrophil-derived chemokines promoting neutrophil and monocyte chemotaxis. Collectively, our data show a differential regulation of cytokine and chemokine expression in human neutrophils by *F. alocis*, *P. stomatis,* and *P. gingivalis*. Deciphering the molecular mechanisms induced by these oral pathogens that modulate the cytokine and chemokine response by human neutrophils is an area of current investigation in our laboratory. 

The release of neutrophil-derived inflammatory mediators will have an important impact on modulation of inflammation in periodontal tissues. In summary, the results from the present study indicate that the three oral pathogens, *P. gingivalis*, *P. stomatis,* and *F. alocis*, manipulate neutrophil effector functions through different mechanisms to promote neutrophil recruitment and perpetuate periodontal inflammation. 

## 4. Materials and Methods 

*Human neutrophil and monocyte isolation.* Blood was drawn from healthy donors and neutrophils were purified using plasma-Percoll gradients as we previously described [39] and in accordance with the guidelines approved by the Institutional Review Board of the University of Louisville. The purity check of the isolated cell fraction determined by microscopic evaluation showed that ≥ 95% of the cells were neutrophils. Cell viability was confirmed by trypan blue exclusion indicated that ≥ 97% of cells were viable. For the experiments related to mRNA extraction and for supernatant collection after bacterial challenge to measure neutrophil-derived cytokines and chemokines highly purified neutrophils were used. For these assays, neutrophils obtained after plasma-Percoll gradient were further purified using the negative selection EasySep Magnet human neutrophil enrichment kit (Stemcell technology Vancouver, BC, Canada) as we previously described [40]. 

Peripheral blood mononuclear cells (PBMCs) fraction obtained after the plasma-Percoll gradient was washes twice in Krebs+, counted and plated into 6 well plates and allow the monocytes to attach to the surface for 2 h at 37 °C and 5% CO_2_ incubator as we previously described [41]. 

*Bacterial growth conditions. F. alocis* ATCC 38596 was cultured in brain heart infusion (BHI) broth supplemented 20 mg/mL yeast extract, L-cysteine (0.1%) and arginine (0.05%) for 14 days anaerobically at 37 °C as previously described [13,15]. *P. stomatis* strain CM2 was cultured anaerobically at 37 °C in Tryptic Soy Broth supplemented with 20 mg/mL yeast extract, 1% hemin and 1% reducing agent as previously described [22]. *P. gingivalis* ATCC 33277 was cultured anaerobically at 37 °C in trypticase soy broth supplemented with yeast extract (1 mg/mL), hemin (5 µg/mL) and menadione (1 µg/mL).

*Reverse transcription and quantitative real-time PCR (RT-qPCR).* Highly purified human neutrophils (10–20 × 10^6^ cells/mL) were unstimulated (basal), stimulated with *F. alocis* at a multiplicity of infection of 10 bacteria per neutrophil. Total RNA was extracted at 1 h, 3 h, 6 h, and 24 h post bacterial challenge by the hybrid method. Neutrophils were lysed using Trizol (Life Technologies, Carlsbad, California) followed by RNeasy Mini Kit (Qiagen, Venlo, Neitherland) from aqueous phase loading on column, according to the manufacturer’s instructions. Purified total RNA was then reverse-transcribed into cDNA using High capacity RNA to cDNA kit (Applied biosystem, Foster City, California), while qPCR was carried out using SYBR® Green PCR Master Mix (Applied Biosystem, Foster City, California) on an Applied Biosystems StepOne Plus cycler with stepone software V2.2.2. Sequences of the gene-specific primers (Integrated DNA Technologies, Skokie, Illinois) used in this study are listed in Table 2. Data were calculated and expressed as mean normalized expression (MNE) units after GAPDH normalization as previously described [42].

*Cytokine and chemokine production.* Highly purified neutrophils (2.5 × 10^6^ cells/0.5 ml) were resuspended in colorless RPMI medium (Sigma-Adrich, St. Louis, MO) supplemented with 5% pooled human serum (Sigma-Aldrich, St. Louis, MO) and plated in 24 well plates. Cells were left unstimulated (basal), or stimulated with serum opsonized *F. alocis* (MOI 10), non-opsonized-*F. alocis* (MOI 10), *P. stomatis* (MOI 10), *P. gingivalis* (MOI 10). Bacterial phagocytosis was synchronized by centrifugation at 600 g for 4 min at 14 °C. After the centrifugation step the plates were transferred to the tissue culture incubator for 24 h. Bacteria- and cell-free supernatants were collected and stored at −80 °C until used. Levels of TNFα, IL-1β and CXCL8 were measured by ELISA (eBioscience-Thermo Fisher Scientific, Waltham, MA). Levels of IL-1ra, CXCL1, CCL2, CCL3, and CCL4 were measured by Milliplex assays (Millipore-Sigma, Billerica, MA). 

For TLR2/1 and TLR2/6 assays, human embryonic kidney cells (HEK293) stably transfected with plasmid containing human TLR2/1 or TLR2/6 under the control of an IL-8 (CXCL8) promoter were used. Cells were cultured in DMEM medium (Thermo Fisher Scientific, Waltham, MA), with 1 mM sodium pyruvate, 50 units/mL penicillin, 50 mg/mL streptomycin, 10% heat-inactivated FBS (Atlanta Biologicals Inc, Flowery Branch, GA). HEK293-TLR2/1 cells were plated at 0.4 × 10^6^ cells/well on a 24 well-plate and left unstimulated (basal), stimulated with increasing concentrations of the agonist Pam_3_CSK_4_ from 0.01 up to 10 µg/mL (InvivoGen, San Diego, CA), increasing doses of *F. alocis* (MOI 1 up to 100), increasing doses of *P. stomatis* (MOI 1 up to 100) for 24 hr. at 37 °C in 5% CO_2_. HEK293-TLR2/6 cells were plated at 0.4 x 10^6^ cells/well on a 24 well-plate and left unstimulated (basal), stimulated with increasing concentrations of the agonist FSL1 from 0.5 up to 1000 ng/mL (InvivoGen, San Diego, CA), and the same doses of the oral bacteria were used as described for the TLR2/1 cells. Cell-free supernatants were collected and levels of CXCL-8 measured by ELISA (eBioscience-Thermo Fisher Scientific, Waltham, MA) following the manufactures instructions. 

*Neutrophil and monocyte chemotaxis assays.* Freshly isolated neutrophils (4 × 10^5^ cells/0.1 mL) or monocytes (4 × 10^5^ cells/0.1 mL) were added to the upper chamber of the transwell inserts contained in 24 well plates (VWR, Corning). For neutrophil chemotaxis a pore size of 3 µm polycarbonate membrane was used, and for monocytes chemotaxis an 8 µm pore size membrane was used. Chemotaxis was initiated by adding 600 µL of Krebs-Ringer phosphate buffer (pH 7.2) containing 0.2% dextrose (Krebs), or different chemoattractants into the lower chamber. The chemoattractants used were CXCL1 (10 nM, Sigma), CCL3 (100 ng/mL, Sigma), along with the bacteria-cell-free supernatants collected from unstimulated, *F. alocis*-challenged neutrophils (MOI 10, 24 h), *P. stomatis*-challenged neutrophils (MOI 10, 24 h), *P. gingivalis*-challenged neutrophils (MOI 10, 24 h). Bacteria were removed from the collected supernatants by passing through a sterile 0.2 µm filter. After 30 or 120 min the transwell membranes were stained with a HEMA 3 stain set kit following the manufacturer’s instructions (Thermo Fisher Scientific). Chemotaxis was assessed by light microscopic (VWR Compound Trinocular Microscope) examination (magnification x100) of the underside of the membrane. The average number of cells from a total of 10 fields was determined and data were normalized by the area of membrane circle and field of view as previously described [26]. 

## Figures and Tables

**Table 1:** Cytokine and chemokine production by *F. alocis*-stimulated human neutrophils.

Cytokines	Basal	Non-op-*F. alocis*	Op-*F. alocis*
IL-1β (pg/0.5 × 10^6^ cells)	1.30 ± 0.736	5.125 ± 0.417 **	5.464 ± 0.720 **
TNFα (pg/0.5 × 10^6^ cells)	6.448 ± 1.302	46.759 ± 6.382 **	35.514 ± 7.401 *
IL-1ra (pg/2 × 10^6^ cells)	38.948 ± 7.785	194.527 ± 24.179 ***	138.451 ± 7.678 **
**CXCL Chemokines**			
CXCL1 (ng/2 × 10^6^ cells)	0.004 ± 0.002	0.478 ± 0.078 ***	0.445 ± 0.073 ***
CXCL8 (ng/0.1 × 10^6^ cells)	0.596 ± 0.214	4.492 ± 1.166 *	3.818 ± 0.849 *
**CCL Chemokines**			
CCL3 (pg/2 × 10^6^ cells)	25.087 ± 10.922	108.511 ± 16.580	232.526 ± 80.041 *
CCL4 (pg/2 × 10^6^ cells)	133.315 ± 43.227	639.611 ± 104.467	709.178 ± 203.417 *

Note. Human neutrophils were stimulated with non-opsonized (Non-op) or opsonized (Op) *F. alocis* (MOI 10:1) for 24 h. Cell/bacteria free supernatant were collected and cytokine and chemokine release measured by ELISA (IL-1β, TNFα, CXCL8) or Milliplex Luminex (IL-1ra, CXCL1, CCL3, CCL4). Data are presented as mean ± SEM. IL-1β, CCL3 n = 4; CXCL8, TNFα, CCL4 n = 5; IL-1ra n = 6; CXCL1 n = 7. * *p* < 0.05, ** *p* < 0.01, *** *p* < 0.001 compared to basal.

**Table 2:** Gene specific primers.

Genes		Sequence
TNF-α	Forward	5’CAGCCTCTTCTCCTTCCTGAT3’
	Reverse	5’GCCAGAGGGCTGATTAGAGA3’
IL-1ra	Forward	5’AACTAGTTGCTGGATACTTGCA3’
	Reverse	5’CCAGACTTGACACAGGACAG3’
IL-1b	Forward	5’TACCTGTCCTGCGTGTTGAA3’
	Reverse	5’TCTTTGGGTAATTTTTGGGATCT3’
CXCL1	Forward	5’AACCGAAGTCATAGCCACAC3’
	Reverse	5’CCTCCCTTCTGGTCAGTTG3’
CXCL2	Forward	5’AACCGAAGTCATAGCCACAC3’
	Reverse	5’CTTCTGGTCAGTTGGATTTGC3’
CXCL3	Forward	5’AAGTGTGAATGTAAGGTCCCC3’
	Reverse	5’GTGCTCCCCTTGTTCAGTATC3’
CXCL8	Forward	5’GAGCACTCCATAAGGCACAAA3’
	Reverse	5’ATGGTTCCTTCCGGTGGT3’
CCL3	Forward	5’CGGCAGATTCCACAGAATTTC3’
	Reverse	5’AGGTCGCTGACATATTTCTGG3’
CCL4	Forward	5’TCCTCGCAACTTTGTGGTAG3’
	Reverse	5’TTCAGTTCCAGGTCATACACG3’
GAPDH	Forward	5’CTTTGGTATCGTGGAAGGACTC3’
	Reverse	5’GTAGAGGCAGGGATGATGTTC3’
